# Interleukin-1β More Than Mechanical Loading Induces a Degenerative Phenotype in Human Annulus Fibrosus Cells, Partially Impaired by Anti-Proteolytic Activity of Mesenchymal Stem Cell Secretome

**DOI:** 10.3389/fbioe.2021.802789

**Published:** 2022-01-28

**Authors:** Raquel M. Gonçalves, Taryn Saggese, Zhiyao Yong, Joana R. Ferreira, Anita Ignatius, Hans-Joachim Wilke, Cornelia Neidlinger-Wilke, Graciosa Q. Teixeira

**Affiliations:** ^1^ Institute of Orthopaedic Research and Biomechanics, Trauma Research Centre, Ulm University, Ulm, Germany; ^2^ Instituto de Investigação e Inovação Em Saúde (i3S), Universidade Do Porto, Porto, Portugal; ^3^ Instituto de Engenharia Biomédica (INEB), Universidade Do Porto, Porto, Portugal; ^4^ Instituto de Ciências Biomédicas Abel Salazar (ICBAS), Universidade do Porto, Porto, Portugal

**Keywords:** disc degeneration, disc herniation, annulus fibrosus cells, mechanical loading, inflammation, mesenchymal stem cells, secretome, matrix metalloproteinases

## Abstract

Mesenchymal stem/stromal cell (MSC)–based therapies for low back pain and intervertebral disc (IVD) degeneration have been emerging, despite the poor knowledge of their full mechanism of action. As failure of the annulus fibrosus (AF) is often associated with IVD herniation and inflammation, the objective of the present study was to investigate the impact of the MSC secretome on human AF cells exposed to mechanical loading and a pro-inflammatory environment. Human AF cells isolated from IVD biopsies from patients with adolescent idiopathic scoliosis (AIS) or disc degeneration (DD) were exposed to physiological cyclic tensile strain (CTS) for 72 h in a custom-made device, with or without interleukin (IL)-1β medium supplementation. AF cells stimulated with CTS + IL-1β were then treated with secretome from IL-1β–preconditioned MSCs for 48 h. AF cell metabolic activity, gene expression, protein secretion, matrix metalloproteinase (MMP) activity, and tissue inhibitor of MMPs (TIMP) concentration were evaluated. Expanded AF cells from AIS and DD patients revealed similar metabolic activity and gene expression profiles. CTS stimulation upregulated collagen type I (*COL1A1*) expression, while IL-1β significantly stimulated *IL-6*, *IL-8*, *MMP-1*, and *MMP-3* gene expression and prostaglandin E_2_ production by AF cells but downregulated *COL1A1*. The combination of CTS + IL-1β had a similar outcome as IL-1β alone, accompanied by a significant upregulation of elastin. The MSC secretome did not show any immunomodulatory effect on CTS + IL-1β–stimulated AF cells but significantly decreased *MMP-1*, MMP-2, *MMP-3*, and *MMP-9*, while increasing the production of TIMP-1. The obtained results demonstrate a stronger impact of the inflammatory milieu on human AF cells than upper physiologic mechanical stress. In addition, a new MSC mechanism of action in degenerated IVD consisting of the modulation of AF MMP activity was also evidenced, contributing to the advancement of knowledge in AF tissue metabolism.

## Introduction

Low back pain (LBP) is one of the top 10 disorders in the number of years lived with disability for adolescents and young adults (from 10 to 49 years old), affecting 70–85% of the world population and compromising healthy aging (Vos et al., 2020). LBP can be considered a multifactorial disorder but has been strongly associated with degeneration of the intervertebral disc (IVD). Current therapies include physical therapy or anti-inflammatory drugs, and if LBP does not improve, invasive surgery such as discectomy, spinal fusion, or IVD prosthesis can be used (Maher et al., 2017). Such treatments may alleviate LBP symptoms but reduce patients’ mobility, might be temporary, and, overall, do not target the underlying problem.

The outer ring of the IVD, the annulus fibrosus (AF), consists of concentric collagen fibers interconnected by a network of elastin and fibrillin, interrupted at distinct locations by a zone that links the different lamellae, known as the translamellar bridging network (TBLN). AF failure is often associated with structural weakness in restraining the hydrated inner structure, the nucleus pulposus (NP). Factors that induce AF failure include mechanical stress, which has been previously shown to increase pro-inflammatory gene expression in human AF cells (Pratsinis et al., 2016). Mechanical loading appears to act synergistically with a pro-inflammatory environment, weakening the AF through TBLN deregulation (Saggese et al., 2019). But moderate mechanical stress has also shown to rescue pro-inflammatory gene expression of AF cells exposed to a pro-inflammatory stimulus (Zhang et al., 2021). Overall, the pathomechanism of AF mechanical weakening and consequent disc herniation remains to be fully understood.

IVD herniation involves alterations in the AF but also in the NP, including loss of proteoglycans and water content, upregulation of matrix metalloproteinases (MMP-1, -2, -3, -9, among others) and inflammatory mediators (tissue necrosis factor (TNF)-α, interleukin (IL)-1β, etc) that contribute to the weakening and mechanical failure of the disc tissue (Risbud and Shapiro, 2014). More recently, the complement activation product terminal complement complex (TCC), an inflammatory trigger, has also been suggested to be involved in the disease progression (Teixeira et al., 2021a). Unbalanced production of MMPs weakens the IVD extracellular matrix (ECM), which can lead to NP extrusion through the AF, ultimately leading to lumbar disc herniation (LDH). LDH accounts only for 5–15% of LBP cases but can induce sciatica symptoms and loss of bladder/bowel control and is the main cause of spine surgery (Weinstein et al., 2008). Moreover, about 18% of patients present signs of IVD re-herniation after surgery (Shin et al., 2019). Early treatment to reinforce AF in LDH would be an important strategy to prevent disease progression.

Cell-based therapies are emerging to treat LBP and IVD degeneration. In particular, mesenchymal stem/stromal cell (MSC)–based therapies have been shown to promote ECM synthesis in NP and increase disc height in small animal models (Sakai et al., 2006). MSC intravenous administration has also shown to reduce IVD herniation in a rat model of punctured IVD (Cunha et al., 2017). In the clinical trials conducted so far, MSC transplantation appears to be safe and patients reported less pain, but no significant alterations in the IVD were observed (Orozco et al., 2011; Noriega et al., 2017). This discrepancy between animals and humans might be due to the fact that the former lacks the biped position of the human spine and retain a large population of notochordal cells, thus a higher regenerative capacity. In a pro-inflammatory environment characteristic of the degenerated IVD, we have demonstrated that MSCs have been shown to have immunomodulatory features, and we hypothesize that this may occur via a paracrine mechanism (Teixeira et al., 2018). Recently, we have shown that MSC secretome, obtained upon IL-1β preconditioning, also reduces pro-inflammatory markers in both NP and AF organ cultures (Ferreira et al., 2021; Neidlinger-Wilke et al., 2021). Nevertheless, as these studies were conducted in a bovine model, the direct clinical translation may have some restraints. In fact, MSCs and their secretome can be highly influenced by the cells’ microenvironment, as reviewed by Ferreira et al. (2018). The degenerated IVD is a harsh biochemical and biomechanical condition, with acidic pH, high concentration of pro-inflammatory factors, and matrix-degrading enzymes, under high and complex mechanical loading. This environment is a challenge for the successful translation of cell therapies, including those with MSCs. Additionally, more complex *in vitro* studies are needed which take the multifaceted IVD environment and the interaction of the different influencing factors into account. In this study, we hypothesized that 1) a synergistic action between mechanical loading and a pro-inflammatory microenvironment can deregulate human AF cell phenotypes, and 2) the MSC secretome has an immunomodulatory impact on human AF cell metabolism.

## Materials and Methods

### MSC Expansion and Secretome Production

Human MSCs (Lonza, *n* = 3, donor information is summarized in Supplementary Table S1) were seeded (3,000 cells/cm^2^) and routinely expanded in MSC medium composed of low-glucose Dulbecco’s modified Eagle medium (DMEM, Gibco) supplemented with 10% fetal bovine serum (FBS, HyClone), 1% penicillin/streptomycin (10,000 U/mL—10 mg/ml, Gibco), and 0.5% amphotericin B (250 μg/ml, Gibco), at 37°C, 21% oxygen and humidified atmosphere. The medium was exchanged twice a week, and cells were trypsinized when reaching 70% confluency. For secretome production, 1x10^6^ MSCs (passage 5–9) were seeded in 6-well plates (Corning) and incubated in 5 ml of MSC medium supplemented with recombinant human IL-1β (10 ng/ml, R&D Systems), at 37°C in a humidified atmosphere with 6% O_2_ and 8.5% CO_2_ for 48 h, as previously optimized for MSC preconditioning (Ferreira et al., 2021). MSC secretome (MSCsec) was collected by centrifugation at 1800 *g* for 5 min, at 4°C, to remove cell debris and then stored at −80°C until further use.

### Human AF Cell Isolation and Culture

Human AF cells were isolated from a total of 12 patients undergoing surgery in the lumbar region: six samples from patients with adolescent idiopathic scoliosis (AIS, age: 16 ± 2 years old, 4 males/2 females), and six samples from patients with disc degeneration (DD, age: 65 ± 11 years old, 5 males/1 female, Pfirrmann grades IV-V). Detailed patient information is summarized in Supplementary Table S2. Open surgery was performed to remove the disc tissue. The AF tissue was macroscopically separated from the cartilaginous endplate and NP, as previously described (Teixeira et al., 2021a; Teixeira et al., 2021b). Visibly fibrous tissue with a clearly identifiable lamellar structure was selected for AF cell isolation. AF cells were obtained upon enzymatic digestion of the tissue for 3–5 h in 0.8 mg/ml collagenase type II (Sigma-Aldrich) in IVD medium without FBS: low-glucose DMEM (Gibco) supplemented with 1% non-essential amino acids (Biochrom), 1% penicillin/streptomycin, 5% amphotericin B, and 1.5% 5M NaCl/0.4 M KCl solution (to adjust osmolarity to 400 mOsm), under agitation, reduced oxygen supply (6% O2 and 8.5% CO2), 37°C, and saturated humidity. Cells were then expanded under sterile conditions in IVD medium containing 5% FBS Superior (Biochrom), as previously described (Teixeira et al., 2016).

### Stimulation of AF Cells With CTS and IL-1β and Treatment With MSC Secretome

**FIGURE 1:**
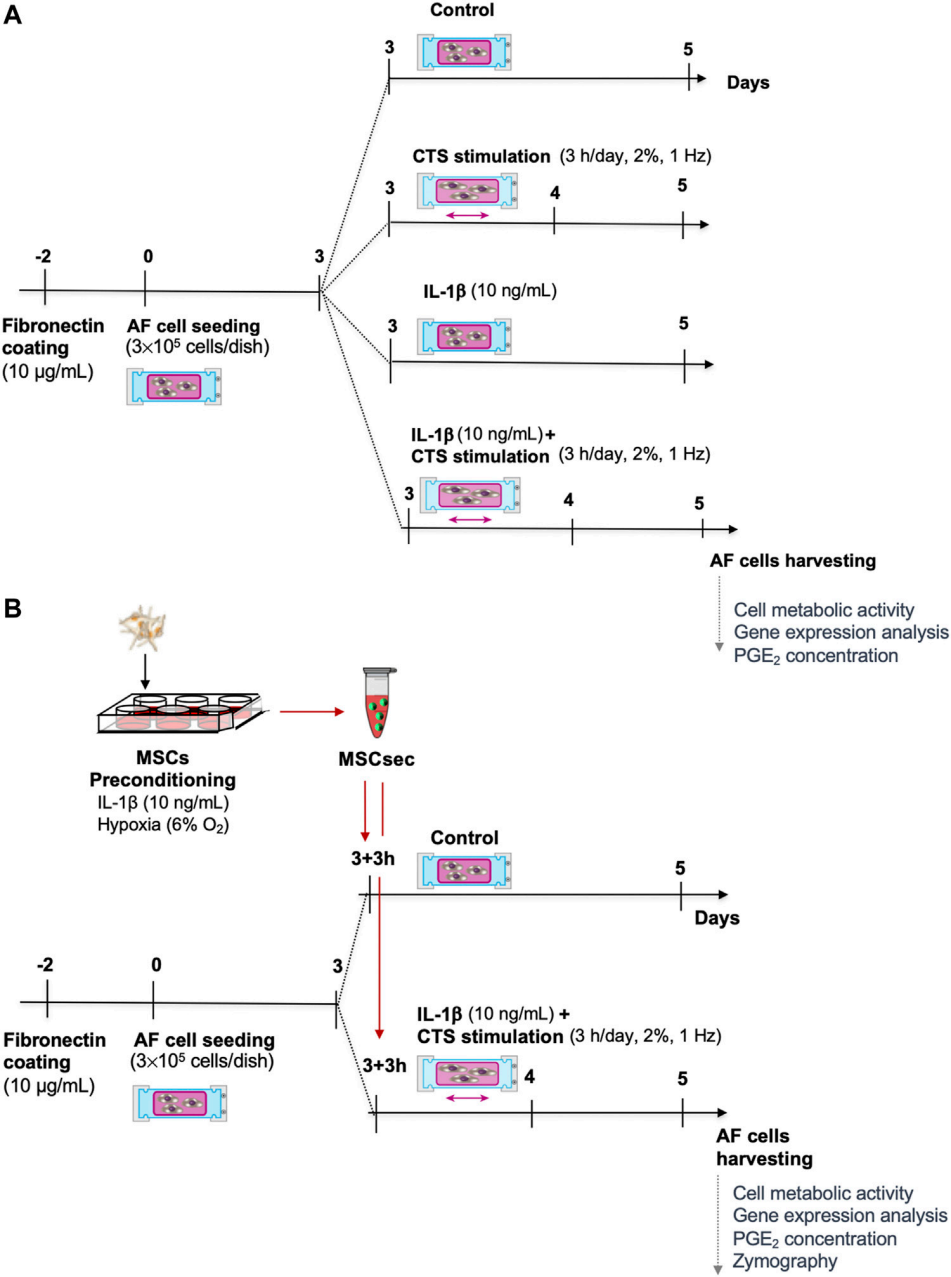
Experimental timeline. **(A)** Human annulus fibrosus (AF) cells were cultured in a custom-made electromechanical device for the application of cyclic tensile strain (CTS) to deformable silicone dishes, with IL-1β (10 ng/ml) or with CTS + IL-1β. **(B)** The CTS + IL-1β–stimulated AF cells were treated with secretome from human bone marrow–derived mesenchymal stem/stromal cells (MSC) upon preconditioning with IL-1β for 48 h.

Human AF cells (passages 3 to 4) were seeded in silicone dishes (300,000 cells/dish) coated with fibronectin (10 μg/ml) and placed in a custom-made cyclic tensile strain (CTS) device (Neidlinger-Wilke et al., 1994), previously modified as described (Pratsinis et al., 2016). After 3 days, the medium was exchanged, and the AF cells were stimulated for 48 h as follows: 1) 2% CTS at 1 Hz was applied 3 h/day to the silicone dishes, similar to what was previously optimized (Wuertz et al., 2007); 2) IL-1β (10 ng/ml), as previously seen to induce a pro-inflammatory response in bovine IVD organ culture (Teixeira et al., 2016); and 3) CTS (1 Hz, 3 h/day) + IL-1β (10 ng/ml). Unstimulated AF cells were kept as a control group. The experimental timeline is depicted in Figure 1A.

In a subsequent set of experiments, the MSC secretome was added to AF cells stimulated with CTS + IL-1β, 3 h after stretching, in a 1:1 ratio with IVD medium. The MSC secretome addition to unstimulated AF cells was used as a control. The experimental timeline is represented in Figure 1B.

After 48 h, AF cells and conditioned medium were collected for different analyses. Conditioned medium was removed and frozen at −80°C for posterior protein analysis by ELISAs and zymography. AF cell metabolic viability was evaluated by resazurin assay, after which cells were immediately shock frozen in RNAlater ICE (Invitrogen) and liquid nitrogen and stored at −80°C for RNA isolation.

### Metabolic Activity of AF Cells

The metabolic activity of AF cells was assessed by resazurin reduction assay. AF cells were incubated with 0.02 mg/ml resazurin sodium salt (Sigma-Aldrich) solution in IVD medium for 3 h at 37°C. Fluorescence intensity was measured in a spectrophotometer microplate reader (Tecan), with 530-nm excitation and 590-nm emission filters.

### Gene Expression Analysis of AF Cells

AF cells frozen in RNAlater ICE were thawed, the RNAlater was removed, and RNA was isolated using an RNeasy Mini kit (Qiagen). RNA concentration and quality were determined by spectrophotometry (Spark, Tecan). For cDNA synthesis with integrated removal of DNA contamination, 1 µg of RNA was treated with a QuantiTect Reverse Transcription kit (ThermoFisher Scientific). Gene expression analysis was performed with primers for the reference gene human glyceraldehyde 3-phosphate dehydrogenase (*GAPDH*), as well as for the target genes in Table 1. The transcribed cDNA was mixed with custom-designed primers and the Platinum SYBR Green qPCR SuperMix-UDG (Invitrogen) and ROX Reference Dye (Invitrogen). The runs were performed in a QuantStudio 3 real-time PCR system (Applied Biosystems). The melt curves were analyzed to confirm the specificity of the reaction and a quantification cycle (Cq) 35 cutoff was used. Relative expression levels were calculated by using the Livak method (using the 2^−ΔΔCt^), where ΔΔCt = ΔCt_(sample of interest)_—ΔCt_(control sample)_ and ΔCt = Ct_(gene of interest)_—Ct_(*GAPDH*)_ (Livak and Schmittgen, 2001).

**TABLE 1:** Human oligonucleotide primers used for qRT-PCR. Primers with shown sequence were custom designed. fw: forward; rev: reverse.

Gene	Sequence (forward and reverse primers)	Product size (bp)
*BAX*	fw: 5′-TGG-AGC-TGC-AGA-GGA-TGA-TTG-3′	98
	rev: 5′-GAA-GTT-GCC-GTC-AGA-AAA-CAT-G-3′	
*CD46*	fw: 5′-GTG AGG AGC CAC CAA CAT TT-3′	177
	rev: 5′-GCG GTC ATC TGA GAC AGG T-3′	
*CD55*	fw: 5′-CAG CAC CACCAC AAA TTG AC-3′	215
	rev: 5′-CTG AAC TGT TGG TGG GAC CT-3′	
*CD59*	fw: 5′-CCG CTT GAG GGA AAA TGA G-3′	130
	rev: 5′-CAG AAA TGG AGT CAC CAG CA-3′	
*COL1A1*	fw: 5′- TGA CCT CAA GAT GTG CCA CT -3′	197
	rev: 5′-ACC AGA CAT CCC TCT TGT CC –3′	
Elastin	fw: 5′- CTG-GCT-TTC-GGA-TTG-TCT-CC-3′	109
	rev: 5′-CGT-TGA-TGA-GGT-CGT-GAG-TC -3′	
Fibrillin-1	fw: 5′-CAC-CTG-TGA-GTG-TAA-TGA-TGG-3′	90
	rev: 5′-TAG-CAC-CTC-TGT-GAA-GCA-3′	
*GAPDH*	fw: 5′-TGC ACC ACC AAC TGC TTA GC-3′	87
	rev: 5′-GGC ATG GAC TGT GGT CAT GAG-3′	
*IL-6*	fw: 5′-AGG AGA CTT GCC TGG TGA AA -3′	188
	rev: 5′-CAG GGG TGG TTA TTG CAT CT -3′	
*IL-8*	fw: 5′-GTG CAG TTTT GCC AAG GAGT-3′	196
	rev: 5′-CTC TGC ACC CAG TTT TCC TT-3′	
*MMP-1*	fw: 5′-ATG CTG AAA CCC TGA AGG TG-3′	234
	rev: 5′-CTG CTT GAC CCT CAG AGA CC-3′	
*MMP-3*	fw: 5′-GGA GAT GCC CAC TTT GAT GAT-3′	187
	rev: 5′-CAT CTT GAG ACA GGC GGA AC-3′	

### Quantification of PGE_2_ and TIMPs in AF Culture Supernatants

The concentration of prostaglandin E_2_ (PGE_2_, Arbor Assays), human TIMP-1, TIMP-2, TIMP-3, and TIMP-4 (RayBiotech) was measured by using enzyme-linked immunosorbent assay (ELISA) kits according to the manufacturers’ instructions in AF supernatants, after 5 days of culture.

### Zymography

The MMP activity of AF cells was investigated by analyzing the conditioned medium through gelatin zymography, as previously described (Cardoso et al., 2014). Briefly, the total protein content in the conditioned medium was determined by bicinchoninic acid assay (Bio-Rad), and 15–20 mg of protein was mixed with sample buffer (10% sodium dodecyl sulfate, 4% sucrose, and 0.03% bromophenol blue in 0.5 M Tris–HCl, pH 6.8) and separated on 10% polyacrylamide gels containing 0.1% gelatin (Sigma-Aldrich) as substrate. After electrophoresis, gels were washed twice with 2% Triton X-100. Gelatin gels were subsequently incubated for 16 h at 37°C in 50 mM Tris–HCl, pH 7.5, and 10 mM CaCl_2_. Gels were stained with 0.1% Coomassie Brilliant Blue R-250 (Sigma-Aldrich), 50% methanol, and 10% acetic acid (Merck). The molecular weight and activity of the MMPs were estimated by densitometric analysis.

### Statistical Analysis

Results are presented as box plots with the median ± interquartile range. Statistical analysis was performed using GraphPad Prism 9 software (GraphPad Software, Inc, La Jolla, CA, United States). Normal distribution data were tested using the D’Agostino–Pearson omnibus normality test. A non-parametric unpaired Mann–Whitney test was used to compare AIS and DD samples. A non-parametric paired Friedman test, followed by Dunn’s multiple comparison test, was used to compare AF cell stimuli and the effect of the MSC secretome. Significance was set at *p* < 0.05.

## Results

### Viability and Inflammatory Profile of Human AF Cells Exposed to Mechanical and Inflammatory Stimuli

To evaluate the effect of mechanical loading and inflammatory stimuli on human AF cells’ inflammatory profile, they were cultured in silicone dishes and stimulated with 1) CTS (2%, 1 Hz, 3 h/day), 2) IL-1β (10 ng/ml), or 3) CTS + IL-1β. Unstimulated AF cells were used as controls (Figure 1A). AF cells from both AIS and DD patients were used indiscriminately. We first screened AF cells from different patients for metabolic activity and gene expression profiles regarding apoptosis (*BAX*), cellular complement regulators (*CD46*, *CD55*, *CD59*), inflammatory markers (*IL-6*, *IL-8*), matrix-degrading enzymes (*MMP-1*, *MMP-3*), and ECM proteins (*COL1A1*, elastin, fibrillin-1). No differences were obtained between the different genes in AF cells from the distinct patient groups (Supplementary Figure S1).

**FIGURE 2:**
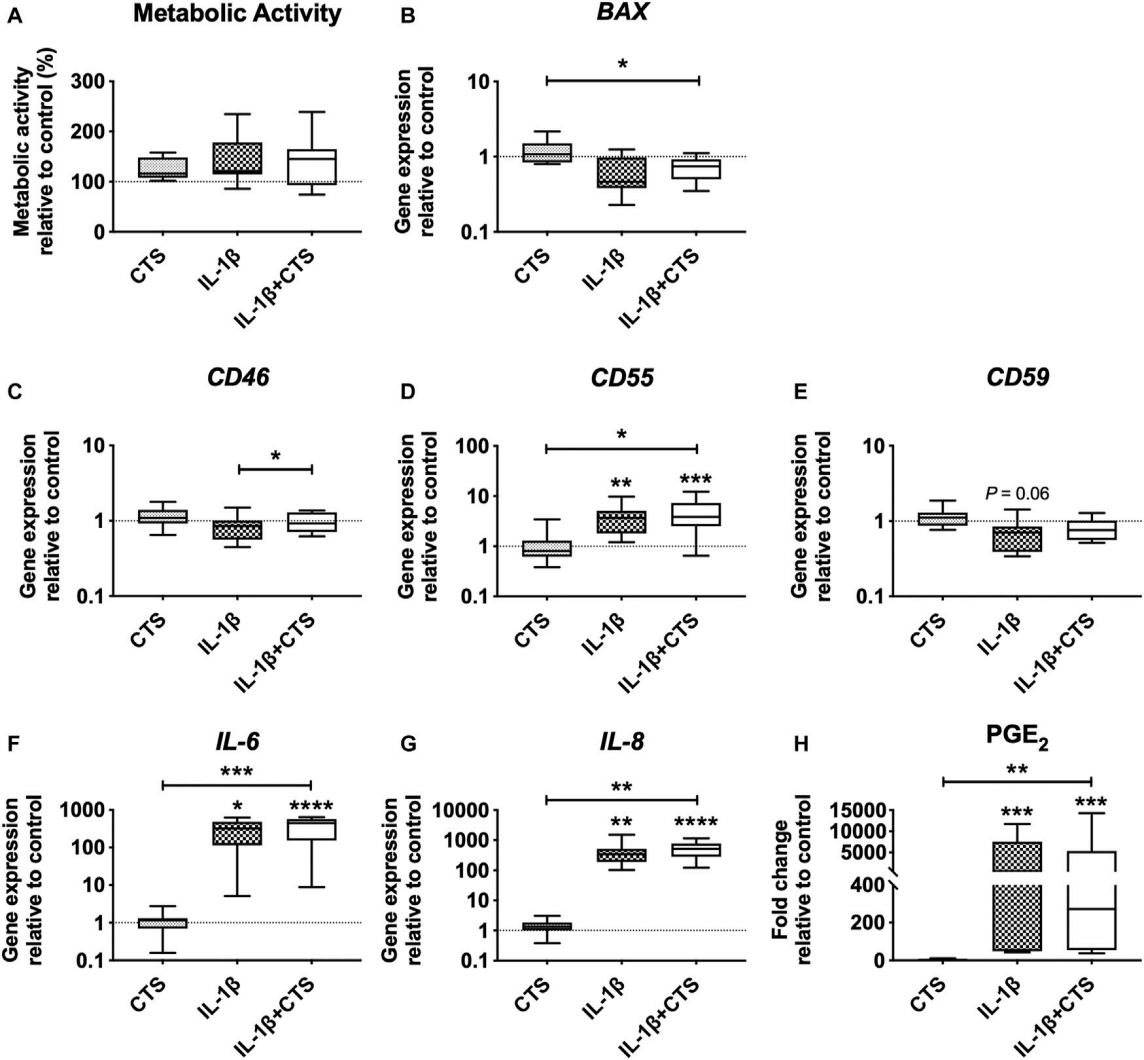
Human annulus fibrosus (AF) cells metabolic activity and gene expression of cell apoptosis marker, complement factors, and pro-inflammatory cytokines, in response to cyclic tensile strain (CTS) and the pro-inflammatory cytokine IL-1β (10 ng/ml) after 48 h. **(A)** AF cells mitochondrial metabolic activity (compared to unstimulated cells). Relative mRNA expression of **(B)** cell apoptosis marker *BAX*, **(C)** complement factors *CD46*, **(D)** *CD55*, and **(E)** *CD59*; pro-inflammatory markers **(F)** *IL-6* and **(G)** *IL-8*. Results were normalized to expression level of *GAPDH* and unstimulated cells. **(H)** Concentration of pro-inflammatory marker PGE_2_ in (fold change to unstimulated cells). *n* = 10, *p < 0.05, **p < 0.01.

To investigate whether AF cell viability was affected by the CTS or IL-1β, mitochondrial metabolic activity and gene expression of *BAX*, an apoptosis regulator member of the *BCL-2* gene family, were analyzed (Figures 2A,B). No differences were observed between the mitochondrial metabolic activity of the different groups. Moreover, no significant differences were observed in the gene expression of *BAX* when comparing CTS, IL-1β and CTS-IL-1β to the unstimulated control group. However, when comparing CTS + IL-1β with the single stimuli CTS and IL-1β, a significant downregulation was found in comparison to CTS (*p < 0.05).

Moreover, CTS + IL-1β stimulation of AF cells significantly increased the expression of the inhibitory complement receptor *CD46*, compared with the IL-1β–stimulated group (*p < 0.05), and of the inhibitor *CD55*, compared with the CTS group (*p < 0.05) and unstimulated cells (***p < 0.001; Figures 2C,D). Interestingly, IL-1β by itself also promoted a significant upregulation of *CD55* (**, *p* < 0.01) and a decrease in *CD59*, the major TCC inhibitor (*p* = 0.06), compared with control cells (Figures 2D,E).

Regarding the expression of inflammatory markers, as expected, IL-1β stimulation induced the upregulation of *IL-6* and *IL-8* gene expression and PGE_2_ secretion by AF cells (*p < 0.05, **p < 0.01 and ***p < 0.001, respectively) in comparison to unstimulated cells (Figures 2F–H). In contrast, CTS stimulation by itself was not shown to affect the production of these markers. The synergic action of mechanical and inflammatory stimuli (CTS + IL-1β) significantly upregulated *IL-6* and *IL-8* expression, compared with both unstimulated (****p < 0.0001) and CTS-stimulated cells (***p < 0.001 for *IL-6* and **p < 0.01 for *IL-8*), and increased PGE_2_ production compared with control (***p < 0.001) and with CTS-stimulated cells (**p < 0.01).

### Matrix Remodeling Capacity of Human AF Cells Exposed to Mechanical and Inflammatory Stimuli

**FIGURE 3:**
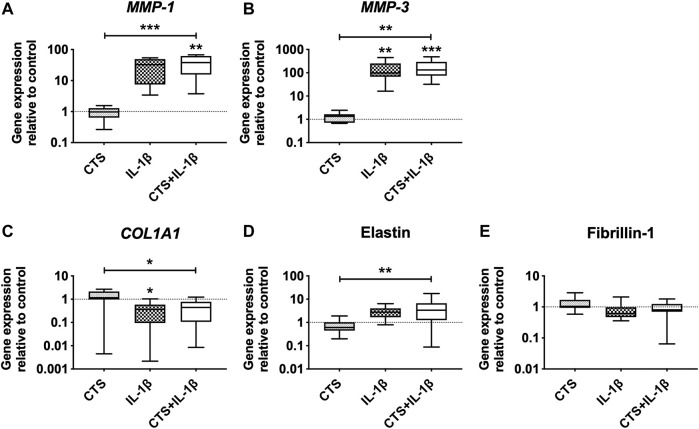
Matrix metalloproteinases (MMPs) and extracellular matrix (ECM) gene expression of human AF cells in response to cyclic tensile strain (CTS) and to pro-inflammatory cytokine IL-1β (10 ng/ml) after 48 h. Relative mRNA expression of matrix-degrading enzymes **(A)** *MMP-1*, **(B)** *MMP-3*, and ECM proteins **(C)** *COL1A1*, **(D)** elastin, and **(E)** fibrillin-1. Results were normalized to expression level of *GAPDH* and unstimulated cells. *n* = 10, *p < 0.05, **p < 0.01.

The effect of mechanical loading and inflammatory stimuli on the production of matrix-degrading enzymes and ECM proteins by human AF cells was also evaluated. The results showed that CTS by itself does not interfere with the matrix remodeling capacity of AF cells. However, when CTS was combined with IL-1β, it significantly increased *MMP-1* and *MMP-3* expression compared with CTS-treatment (***p < 0.001 and **p < 0.01, respectively) and with unstimulated cells (**p < 0.01 and ***p < 0.001, respectively; Figures 3A,B). A significant upregulation of *MMP-3* expression was also observed by the IL-1β-stimulation in comparison to control. In addition, the CTS + IL-1β treatment significantly downregulated *COL1A1* by AF cells (*p < 0.05) and upregulated elastin (**p < 0.01), a key AF protein involved in tissue elasticity, compared to CTS-treated cells (Figures 3C,D). This trend was also observed in the presence of IL-1β only, with *COL1A1* significantly downregulated (*p < 0.05), in comparison with unstimulated cells. In contrast, neither CTS nor IL-1β seemed to affect fibrillin-1, another glycoprotein involved in AF elasticity (Figure 3E).

### MSC Secretome Treatment of Human AF Cells Under Mechanical and Inflammatory Stimuli

Since a synergistic action between mechanical stimuli and a pro-inflammatory microenvironment in human AF cells was observed, we went further and investigated the regulatory effect of the MSC secretome in AF cells in the presence of CTS + IL-1β stimulus. Unstimulated AF cell cultures supplemented with MSC secretome served as controls.

**FIGURE 4:**
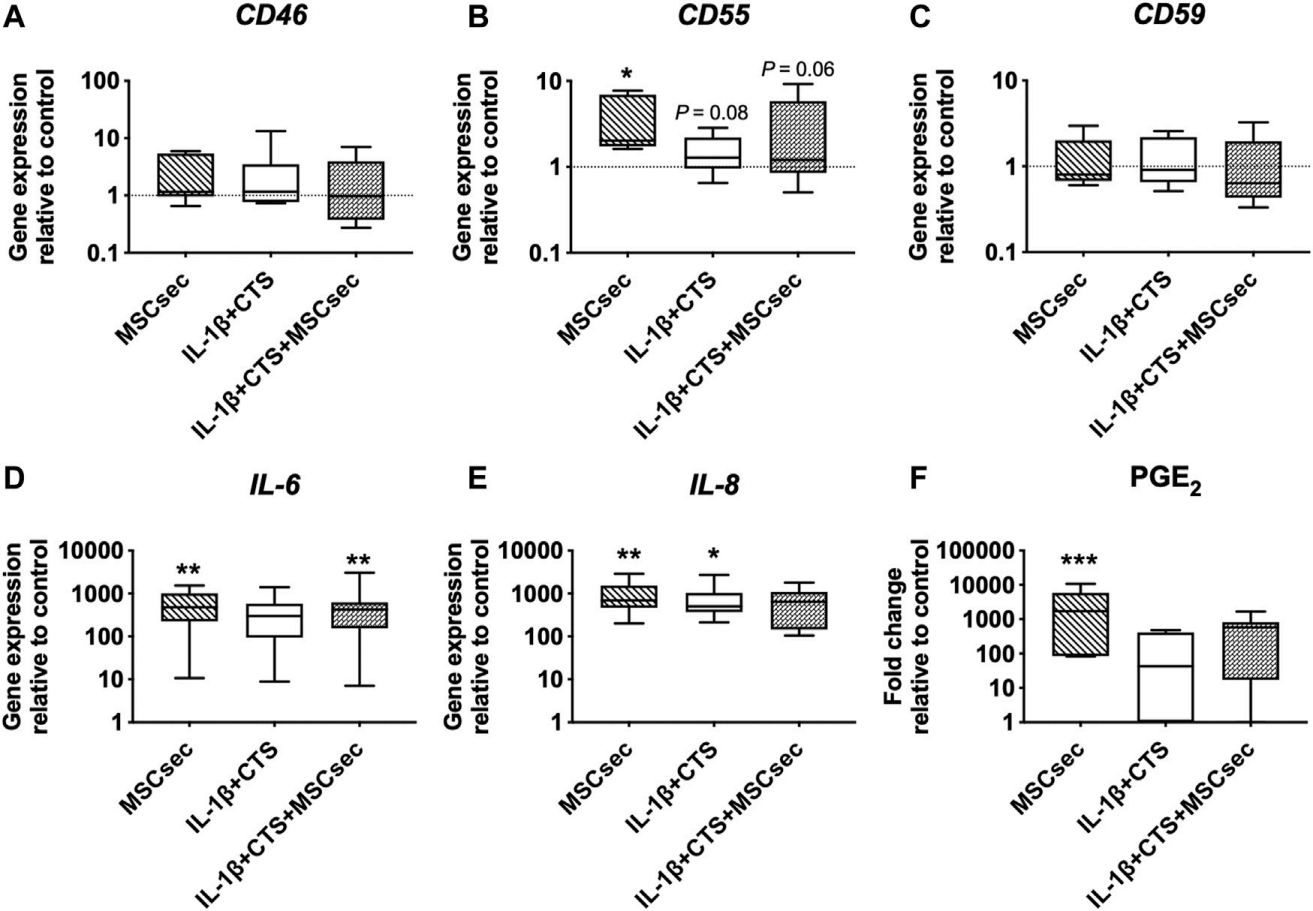
Effect of MSC secretome on human AF cells stimulated with cyclic tensile strain (CTS) and IL-1β (10 ng/ml) in the expression of complement markers and pro-inflammatory cytokines after 48 h. Relative mRNA expression of complement regulatory factors **(A)** *CD46*, **(B)** *CD55*, and **(C)** *CD59*; and pro-inflammatory markers **(D)** *IL-6* and **(E)** *IL-8*. Results were normalized to expression level of *GAPDH* and to unstimulated cells. **(F)** Concentration of pro-inflammatory marker PGE_2_ (fold change to unstimulated cells). *n* = 7, *p < 0.05, **p < 0.01.

**FIGURE 5:**
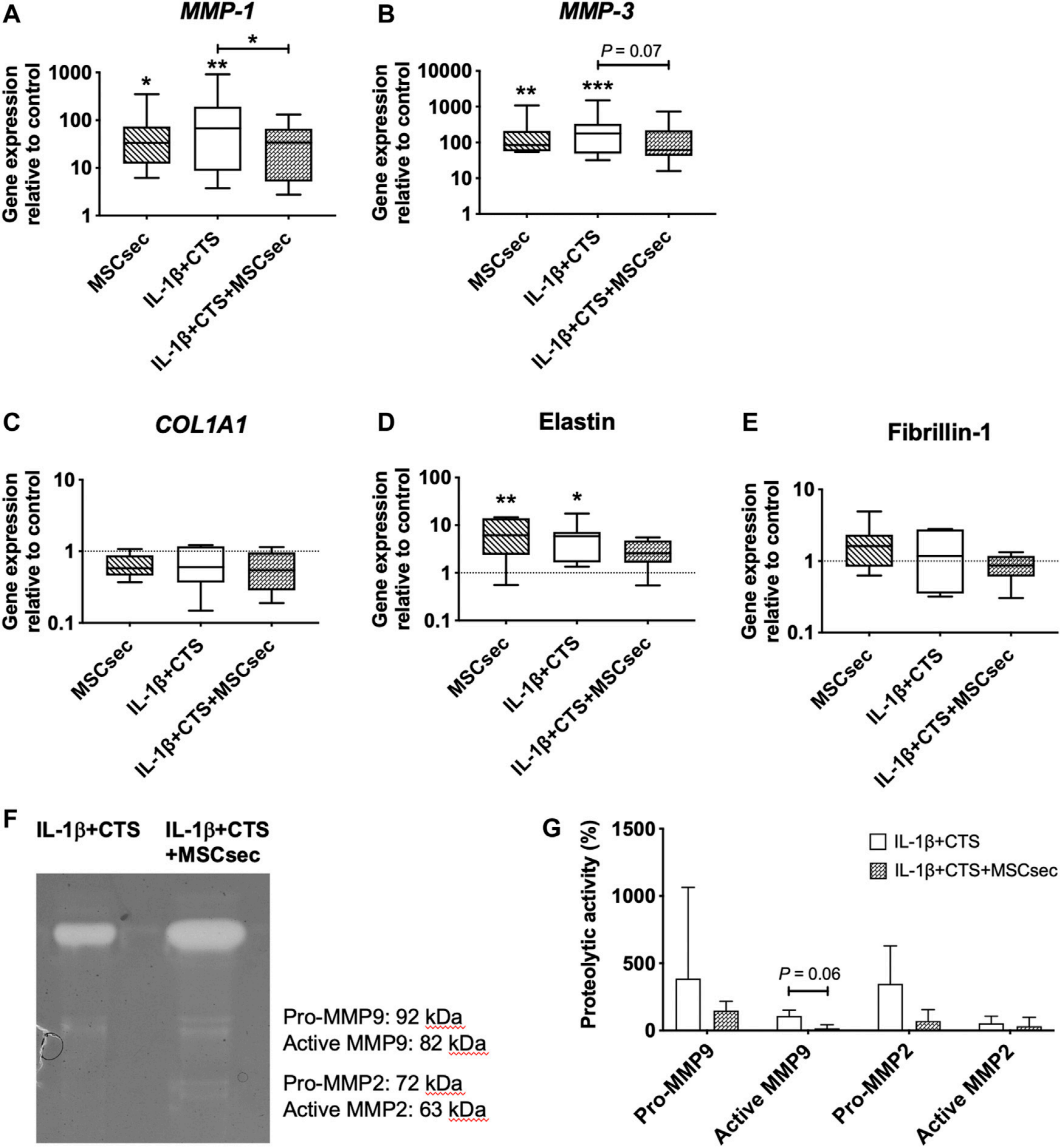
Effect of MSC secretome on human AF cells stimulated with cyclic tensile strain (CTS) and IL-1β (10 ng/ml) in MMPs and ECM proteins after 48 h. Relative mRNA expression of the matrix degrading enzymes **(A)** *MMP-1* and **(B)** *MMP-3*, and of ECM proteins **(C)** *COL1A1*, **(D)** elastin, and **(E)** fibrillin-1. Results were normalized to the expression level of GAPDH and unstimulated cells. *n* = 7, *p < 0.05, **p < 0.01. **(F)** MMP activity in AF cultures media, determined by gelatin zymography **(G)** Quantification of MMP-2 and MMP-9 activity by densitometry (*n* = 3).

The MSC secretome treatment of human AF cells did not affect the gene expression of *CD46* and *CD59* but significantly upregulated *CD55* (*p < 0.05), *IL-6*, and *IL-8* (**p < 0.01) and increased the production of PGE_2_ (***p < 0.001) compared to unstimulated AF cells (Figure 4B, D–F). Regarding the matrix remodeling capacity of human AF cells, the secretome downregulated the gene expression of *MMP-1* (*p < 0.01) and *MMP-3* (*p* = 0.07) (Figures 5A,B) compared to CTS + IL-1β stimulation alone and did not alter *COL1A1*, elastin, or fibrillin-1 expression (Figures 5C–E). In accordance with the increase in pro-inflammatory cytokines, the MSC secretome significantly upregulated *MMP-1* (*p < 0.05) and *MMP-3* (**p < 0.05) gene expression in control AF cells. Furthermore, MMP activity was evaluated by gelatin zymography in AF culture supernatants. The pro- and active forms of the gelatinases MMP-2 (between 72 and 63 kDa) and MMP-9 (between 82 and 92 kDa) (Cardoso et al., 2014), particularly the latter, were identified (Figure 5F). The MMP bands were quantified, and a band of MSC secretome as a control was subtracted from the other conditions tested. The results show that the MSC secretome was able to reduce the activity of gelatin-degrading enzymes produced by human AF cells, namely, the active form of MMP-9 (*p* = 0.06; Figure 5G).

**FIGURE 6:**
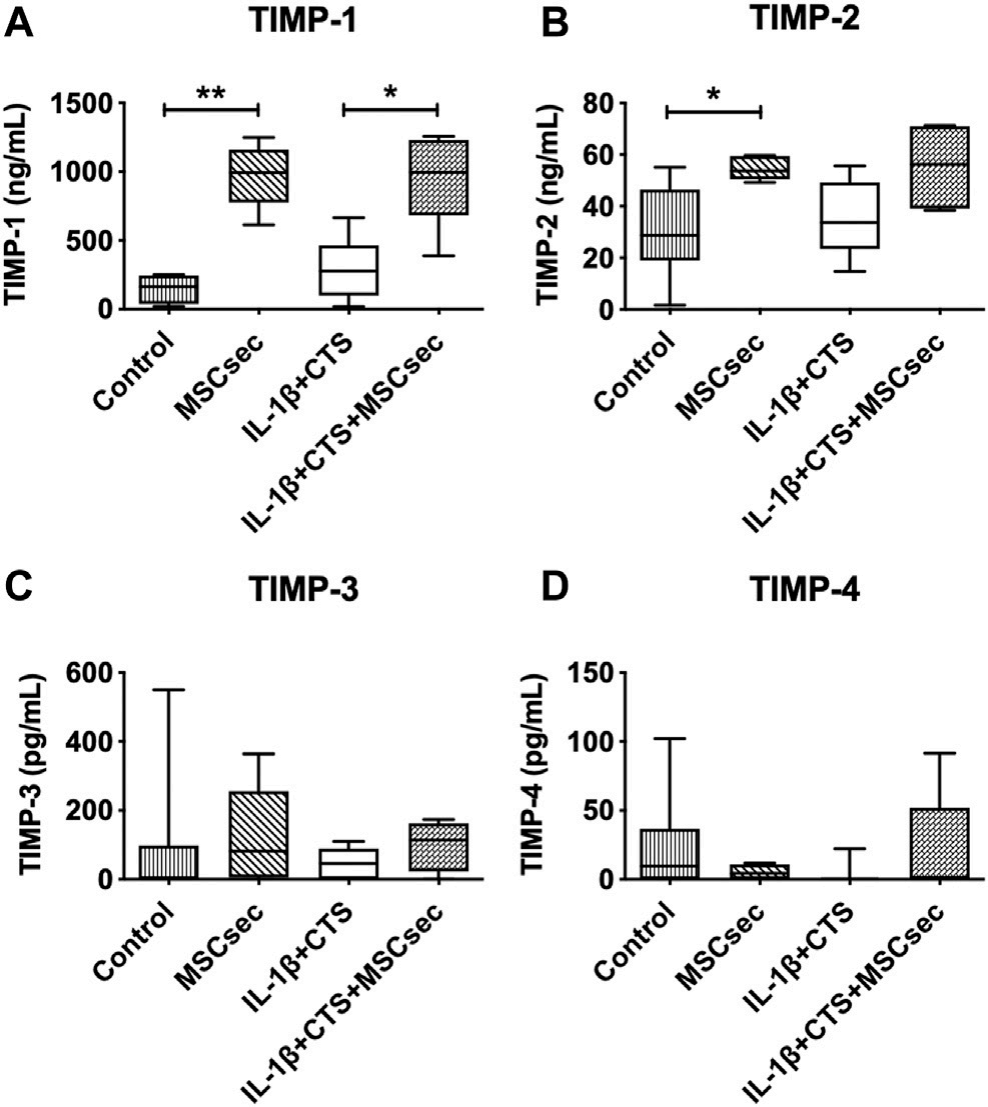
Concentration of tissue inhibitors of metalloproteinases (TIMPs) in the supernatant of AF cultures stimulated with cyclic tensile strain (CTS) and IL-1β (10 ng/ml) and treated with MSC secretome after 48 h, determined by ELISA. **(A)** TIMP-1, **(B)** TIMP-2, **(C)** TIMP-3, and **(D)** TIMP-4. *n* = 5–9, *p < 0.05, **p < 0.01.

In addition, the concentration of different tissue inhibitors of MMPs–TIMP-1, TIMP-2, TIMP-3, and TIMP-4—was quantified in all the conditions (Figure 6), with the concentrations found in the MSC secretome being subtracted from the AF culture supernatants. The MSC secretome significantly increased the production of TIMP-1 by CTS + IL-1β–treated AF cells (*p < 0.05) but did not affect TIMP-2, TIMP-3, or TIMP-4. An increase in TIMP-1 and TIMP-2 was observed in unstimulated cells upon treatment with MSC secretome (**p < 0.01 and *p < 0.05).

## Discussion

In the present study, a pro-inflammatory/catabolic IVD microenvironment was simulated in human AF cell cultures, based on previous *ex vivo* models of bovine NP (Teixeira et al., 2016) and AF (Saggese et al., 2019) organ cultures, which were maintained in low-glucose, iso-osmotic (400 mOsm), and pro-inflammatory conditions with low oxygen supply. In the AF organ cultures, a synergistic action of cyclic tensile strain (CTS) and IL-1β was shown to increase the synthesis of pro-inflammatory mediators and MMP-3 in the AF and to decrease the strength of the tissue (Saggese et al., 2019). In the present study, we evaluated the effects of these stimuli (CTS, IL-1β, and CTS + IL-1β) on the metabolism of human AF cells, as well as possible alterations induced by the treatment with secretome produced by IL-1β–preconditioned MSCs.

For that, AF cells were isolated from patients diagnosed with either AIS or DD and expanded up to passage 3–4, in order to obtain enough cells to culture under different stimuli. Although organ culture models could better mimic the degenerative 3D environment of the IVD, these are difficult to conduct under standardized and reproducible conditions using limited human IVD biopsies, which are small in size and often very fragmented. Therefore, they were used for cell isolation and 2D cultures of AF cells. Despite the differences observed in IVD tissues from disc degeneration compared to AIS patients, namely, increased TCC deposition and expression of *CD59* (a direct TCC inhibitor) (Teixeira et al., 2021a), the expanded AF cells from both patient groups revealed similar behavior. Primary cells always show a certain variability; however, we expected to see differences between the gene expression profiles of cells from AIS and DD due to the lower age of AIS versus DD patients (an average of 16 ± 2 versus 65 ± 11 years old, respectively) and less degeneration in AIS tissues. However, CTS and/or IL-1β did not induce a significantly different response between the two groups for cells in passages 3 to 4. After expansion in 2D cultures, we hypothesize that the cells from DD patients lose their disease phenotype, behaving similarly to AF cells from AIS patients. This suggests that *in vitro* expansion of AF cells might attenuate cellular changes associated with patients’ age and specific disease, in line with recent findings showing similar expression of complement-related markers by AF cells from AIS and DD patients after challenging with IL-1β and cathepsin-D (Teixeira et al., 2021b).

CTS + IL-1β stimulation of human AF cells induced an upregulation of pro-inflammatory cytokines (IL-6, IL-8, and PGE_2_) and MMPs (MMP-1, MMP-3), which was observed to a lesser extent in the presence of IL-1β, but not after CTS stimulation by itself. In the present work, 2% strain alone did not seem to be a strong enough stimulus to induce changes in the AF cell gene expression profile. This might be due to the 2D cell cultures, in contrast to previous investigations of human AF cells cultured in 3D collagen gels, in which differences were observed (Neidlinger-Wilke et al., 2005). CTS stimulation of AF cells has been shown to increase *COL2A1* and aggrecan expression and decrease *MMP-3* expression in human AF cells cultured in 3D collagen gels, independently of the strain magnitude (1–8% strain, 1 Hz) (Neidlinger-Wilke et al., 2005). CTS was also shown to promote upregulation of pro-inflammatory genes, like *COX-2*, *IL-6*, and *IL-8* in AF cells (8% strain, 1 Hz) (Pratsinis et al., 2016). Under mild pro-inflammatory stimulation (1 ng/ml IL-1β) and 6% CTS (0.05 Hz), CTS was suggested to have a protective effect on rat AF cells (Sowa and Agarwal, 2008). Since a role for complement-associated factors has recently been suggested in a degenerative/pro-inflammatory environment, we investigated the gene expression of the cellular complement regulators *CD46*, *CD55*, and *CD59* by human AF cells. Here, CD55, but not the other complement factors, was upregulated by CTS + IL-1β stimulation of AF cells. In bovine AF organ cultures, CTS + IL-1β simulation induced upregulation of *CD46*, but not of *CD55* nor *CD59* (Neidlinger-Wilke et al., 2021). IL-1β stimulation of human articular chondrocytes upregulated *CD45*, *CD55* and *CD59* (Hyc et al., 2003). This indicates that the complement cascade is activated by the CTS and IL-1β stimuli; however, different molecules may be regulated at different timepoints.

Regarding matrix metabolism, CTS + IL-1β stimulation of human AF cells did not alter *COL1A1* and fibrillin-1 production but seemed to enhance elastin synthesis. The latter was also promoted by CTS and IL-1β individually. Fibrillin-1 production was previously shown to be increased within the translamellar bridging-network of bovine AF organ cultures in the presence of IL-1β but not in the presence of CTS + IL-1β (Saggese et al., 2019). Besides the differences between 2D and 3D (organ culture), this study also used lower IL-1β concentration (1 ng/ml), which could explain the different results of the present study. Concerning elastin, TNF-α stimulation of human tenocytes was shown to reduce COL1 deposition while increased elastin gene expression (John et al., 2010), in agreement to what was observed with AF cells in our study. Moreover, it has been described that elastin increases with IVD degeneration and aging, particularly in the AF, consisting in a response to restore the lamellar structure under radial loads that potentially cause delamination (Cloyd and Elliott, 2007).

Previously, we have demonstrated that MSCs cultured with bovine NP in the presence of IL-1β downregulated the pro-inflammatory markers *bIL-6*, *bIL-8*, and *bTNF-α* by NP cells but did not promote ECM synthesis (Teixeira et al., 2018). MSCs secrete numerous soluble factors in response to microenvironmental cues, which include pro-inflammatory stimulation, 3D environment, or hypoxia. Therefore, the MSC secretome has been explored as a cell-based/cell-free therapy for several disorders (Ferreira et al., 2018). MSC IL-1β preconditioning has been shown to upregulate chemokines (CCL5, CCL20, CXCL1, CXCL3, CXCL5, CXCL6, CXCL10, CXCL11, and CX (3)CL1), interleukins (IL-6, IL-8, IL-23A, IL-32), toll-like receptors (TLR2, TLR4, CLDN1), MMPs (MMP-1, MMP-3), growth factors (CSF2, TNF-α), and adhesion molecules (ICAM1, ICAM4), compared to unstimulated MSCs (Carrero et al., 2012). Moreover, although the cell passage can affect the cellular response, it has been reported that the composition of the MSC secretome does not vary significantly between passages 3 and 12 (Serra et al., 2018).

In the IVD, the MSC secretome has been suggested to stimulate the cellular repair capacity (Brisby et al., 2013). For instance, the secretome of human umbilical cord–derived MSCs was shown to restore the stemness of NP progenitor cells, thus rejuvenating the degenerated NP (Zeng et al., 2020). Moreover, the secretome of IL-1β–preconditioned MSCs, enriched in IL-6, RANTES, MCP-1, IL-1β, TIMP-2, IL-8, GRO α/β/γ, leptin, MIP-3α, and CXCL1, downregulated the expression/production of inflammatory markers by bovine NP, while promoting aggrecan deposition (Ferreira et al., 2021). It was also shown to downregulate the expression/production of inflammatory markers, complement system regulators, MMPs, TIMPs, and collagen by bovine AF cells in an *ex vivo* model (Neidlinger-Wilke et al., 2021). In contrast to the previous findings, in the present study, the MSC secretome did not downregulate the expression of cellular complement regulators and pro-inflammatory cytokines of CTS + IL-1β–stimulated human AF cells but strongly downregulated MMP-1 and MMP-3 gene expression. Furthermore, in this study, it was demonstrated to have reduced MMP-2 and MMP-9 activity of human AF cells in presence of the MSC secretome. MMP-1 and MMP-3 are strongly expressed in the IVD of young adults but not in those of children and adolescents, while MMP-2 and MMP-9 are present in older adults, but in fewer cells (Weiler et al., 2002). Moreover, static compression and annular puncture of rat caudal discs have been shown to stimulate MMP-2 activity with simultaneous AF lamellar disorganization, linking MMP-2 to local matrix degradation and collagen remodeling (Rastogi et al., 2013). Therefore, the MSC secretome could be an interesting approach to modulate matrix remodeling in the AF, although further studies need to be conducted in human IVD to better understand its impact on the tissue biomechanics.

In addition, besides being enriched in TIMP-2 (Ferreira et al., 2021), the MSC secretome also stimulated the production of TIMP-1 and TIMP-2, but not TIMP-3 and TIMP-4, by human AF cells, which could be directly correlated with MMP inhibition. This is in agreement with what was previously observed by Le Maitre et al. (2004), who described an increase in TIMP-1 and TIMP-2, but not TIMP-3, in degenerated human IVD biopsies. Vo et al. (2013) also described sthe upregulation of TIMP-1 with IVD degeneration, as well as a downregulation of TIMP-3. Overall, mechanical and inflammatory stimuli play a crucial role in the modulation of the expression levels of MMPs, ADAMTs, and TIMPs. Inhibition of MMPs and ADAMTSs has demonstrated efficacy and therapeutic potential in slowing osteoarthritis progression (Fosang and Little, 2008), which suggests that therapies proposing the inhibition of matrix-degrading enzymes or stimulation of TIMPs are promising for IVD, although they should be tissue-specific and carefully localized.

Our study shows a therapeutic effect of the MSC secretome on the AF cells challenged with CTS + IL-1β. To induce a pro-inflammatory microenvironment, 10 ng/ml IL-1β was used, but other inflammatory cytokines are known to be widely expressed in IVD degeneration/herniation, such as TNF-α or IL-6, and should be also addressed in future studies. More recently, Wangler et al. (2021) have shown that conditioned medium from traumatically injured versus degenerated IVD has different proteome contents, so different inflammatory cytokine cocktails should be explored in the future to deepen the knowledge of the therapeutic effect of MSC secretome on IVD. *In vivo* experiments that fully recreate the degenerative IVD microenvironment will prove the regulatory effect of the secretome of IL-1β–preconditioned MSCs on the modulation of the immune system response and IVD degeneration.

## Conclusion

Overall, this study demonstrates that the inflammatory milieu, in synergy with tensile strain, has an impact on human AF cells, promoting upregulation of inflammatory markers, cellular complement regulators, and elastin; increasing MMP activity and TIMP production; and decreasing *COL1A1* expression. Moreover, this study evidences a novel aspect of the mechanism of action of the MSC secretome in degenerated IVD that consists in the modulation of MMP activity and TIMP-1 production by AF cells.

## Data Availability

The original contributions presented in the study are included in the article/Supplementary Material, further inquiries can be directed to the corresponding author.
