# Supplementary material for: Interleukin-1β More Than Mechanical Loading Induces a Degenerative Phenotype in Human Annulus Fibrosus Cells, Partially Impaired by Anti-Proteolytic Activity of Mesenchymal Stem Cell Secretome
Source: Front Bioeng Biotechnol. 2022 Jan 28;9:802789. doi: 10.3389/fbioe.2021.802789 (PMC8831733; doi:10.3389/fbioe.2021.802789)
Supplement: Supplementary file 1 [file DataSheet1.DOCX]

Supplementary Material

# Supplementary Materials and Methods

## Donor information

Human mesenchymal stem/stromal cells (MSC) were purchased from Lonza. In Table S1, donor information is provided.

**Table S1** Human MSC donor information

| Donor | Gender | Age (years) | Cell passage |
| --- | --- | --- | --- |
| **1** | female | 33 | 9 |
| **2** | male | 23 | 7 |
| **3** | male | 22 | 5 |

The diagnosis of the patients that underwent surgery in the lumbar region are detailed in Table S2. A total of 6 patients were assigned as adolescent idiopathic scoliosis (AIS, average age: 16±2 years old, age range: 14 – 21 years old) patients, with no signs of disc degeneration, whereas 6 patients were generally groups as disc degeneration (DD, average age: 63±12 years old, age range: 51 – 82 years old) patients. For patients with magnetic resonance imaging data, Pfirrmann grade and Modic changes were assessed.

**Table S2** IVD tissue donor information

| Patient code | Gender | Age (years) | Pfirrmann grade | Modic changes | Cell passage | Pathologic details |
| --- | --- | --- | --- | --- | --- | --- |
| **Adolescent idiopathic scoliosis (AIS) patients** | | | | | | |
| **1** | Male | 21 | — | — | 3 | Trauma |
| **2** | Male | 16 | 2 | 0 | 2 | “S” shaped idiopathic scoliosis |
| **3** | Male | 15 | 2 | 0 | 4 | Thoracic idiopathic scoliosis |
| **4** | Female | 15 | — | — | 4 | “S” shaped idiopathic scoliosis |
| **5** | Male | 16 | 2 | 0 | 4 | “S” shaped idiopathic scoliosis |
| **6** | Female | 14 | 2 | 0 | 4 | Idiopathic scoliosis to the right |
| **Disc degeneration (DD) patients** | | | | | | |
| **7** | Female | 73 | 4, 5 | 0 | 5 | Connection with TLIF, decompression |
| **8** | Female | 56 | 5 | 1 | 3 | L4/5 spinal canal stenosis |
| **9** | Female | 57 | 5 | 1 | 3 | Degenerative scoliosis |
| **10** | Male | 82 | 5 | 1, 3 | 4 | Degenerative scoliosis |
| **11** | Female | 60 | 3, 4 | 1, 2 | 4 | Spinal canal stenosis |
| **12** | Female | 51 | 3, 5 | 3 | 4 | Low back pain, osteochondrosis |

# Supplementary Results


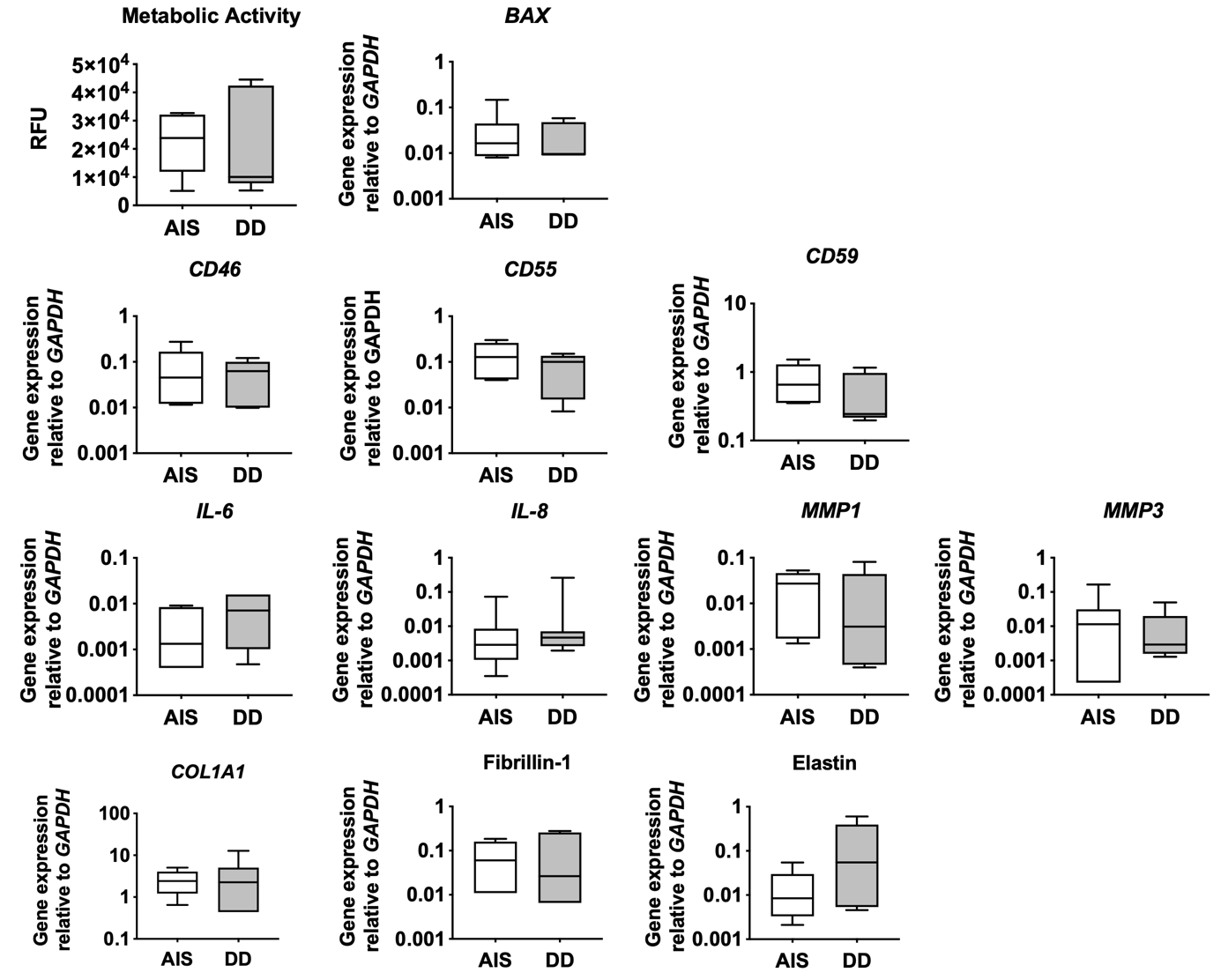


**Supplementary Figure S1.** Viability and gene expression of human AF cells isolated from adolescent idiopathic scoliosis (AIS) and patients with disc degeneration (DD). Cells in passage 3 to 4 (n = 7).
